# Genetic diversity and population structure of barley landraces from Southern Ethiopia’s Gumer district: Utilization for breeding and conservation

**DOI:** 10.1371/journal.pone.0279737

**Published:** 2023-01-05

**Authors:** Hewan Demissie Degu, Tekuamech Fikadu Tehelku, Marie Kalousova, Kazuhiro Sato

**Affiliations:** 1 School of Plant and Horticulture Science, College of Agriculture, Hawassa University, Hawassa, Sidama, Ethiopia; 2 Department of Horticulture Science, College of Agriculture, Wolaita Sodo University, Wolaita Sodo, SNNPR, Ethiopia; 3 Faculty of Tropical AgriSciences, Czech University of Life Sciences Prague, Prague- Suchdol, Czech Republic; 4 Institute of Plant Science and Resources, Okayama University, Kurashiki, Okayama, Japan; Julius Kuhn-Institut, GERMANY

## Abstract

Barley is the fifth most important food crop in Ethiopia. The genetic relationship and population structure studies of barley are limited to gene bank collections. Therefore, this study fills a gap by investigating the selection, consumption, economic value, genetic diversity, and population structure of farm-collected barley from the Gumer district of the Gurage Zone, which has received little attention. The information on the use of barley in the study area was collected using semi-structured interviews and questionnaires. 124 households of 11 kebeles, the smallest community unit, were interviewed. Barley landraces collected were compared with those collected from Japan, the United States (USA), and other Ethiopian locations. Illumina iSelect (50K genotyping platform) was used to identify single nucleotide polymorphisms (SNP) (20,367). Thirty landraces were found in Gumer. Burdaenadenber had the highest on-farm Shannon index estimate (2.0), followed by Aselecha (1.97) and Enjefo (1.95). Aselecha and Fetazer had the highest (44%) and the lowest (29%) richness values, respectively. High and low Simpson index values were found in Aselecha (84%) and Wulbaragenateretero (79%), respectively. The neighbor-joining tree revealed that Gumer landraces formed a separate subcluster with a common ancestral node; a sister subcluster contained barley landraces from Japan. According to the population structure analysis, barley landraces from Gumer differed from Japan and the United States. The principal component analysis revealed that US barley was the most distant group from Gumer barley. The markers’ allele frequencies ranged from 0.10 to 0.50, with an average value of 0.28. The mean values of Nei’s gene diversity (0.38) and the polymorphic information content (0.30) indicated the presence of high genetic diversity in the samples. The clustering of accessions was not based on geographic origin. Significant genetic diversity calls for additional research and analysis of local barley diversity because the selection and use of barley in Ethiopia would have been affected by the preference of ethnic groups.

## Introduction

Barley (*Hordeum vulgare* ssp. *Vulgare*) was domesticated from its wild ancestor *H*. *vulgare* ssp. *Spontanum* [1]. It is the fifth most commonly cultivated crop in Ethiopia [2]. Archeological records indicate that barely appeared in the eighth millennium BC [3]. Currently, it is grown in the highlands of Ethiopia, mainly to produce human food (the grain), animal feed (the straw), and malt for brewing.

The introduction of modern cultivars started in the 20th century in Ethiopia [4]. However, the Ethiopian barley cultivation system still uses several relatively unique landraces [5–9]. Most Ethiopian landraces cluster separately from Asian, European, and North African landraces in a phylogenetic analysis of their genomes [10]. This separation indicates that selection for certain traits affected allele frequency between Ethiopian and non-Ethiopian landraces, leading to linkage disequilibrium. The diversity within these populations is thus indicative of different and genetically diverse landraces, with Ethiopian landraces locally adapted to the traditional farming system and landscape conditions [11].

Despite many collections of barley landraces in the Ethiopian gene bank, the samples lack adequate information. These landraces are sources of germplasm diversity, which would allow future selection and diversification. Geographic information can unravel the domestication process and aid in developing cultivars [10, 12, 13]. Furthermore, it would shed light on the evolutionary relationships of barley around the world [14]. Likewise, barley collection from specific landscapes in Ethiopia could reveal landraces with similar phenotypic characteristics with different genetic backgrounds. These samples could form the basis of an alternative strategy to introduce traits into modern cultivars [15–17].

Understanding and characterizing the cultivated local landraces by researching previously collected samples can help reveal how farmers select specific traits to adapt crops to their local environment [10]. This can provide information on the selection patterns used in different environments. And can be used to improve the adaptability of the barley genome.

Recent studies suggest that barley landraces from multiple locations differentiated into genetically defined populations [11]. Although some studies have identified barley landraces with unique genome structures, it is not being researched extensively. For example, European barley is grouped into three subpopulations based on the sequence of the photoperiod response gene (*Ppd-H1*) [18–20]. Six different haplotypes were found in Asian barley populations, identified by the β-amylase gene (*Bmy1*) at positions 115, 165, 233, 347, and 430, which allows adaptation and selection based on the preference for climate, and food [21–23]. Furthermore, the population structure of barley in different geographic locations indicates multiple selection pressures based on uses and growth environments [1, 6, 14–16].

Single nucleotide polymorphism (SNP) of Illumina iSelect genotyping platforms used to examine barley landraces from the Gumer district, Ethiopia. The objectives of this study were 1) to determine the on-farm diversity of barley in the Gumer district. 2) to determine the genetic diversity and population structure of barley landraces from the Gumer district using SNP markers on the nuclear genome. 3) to compare the pattern of genetic diversity from the United States, Japan, and other Ethiopian barley accessions.

## Materials and methods

### Research area

**Fig 1:**
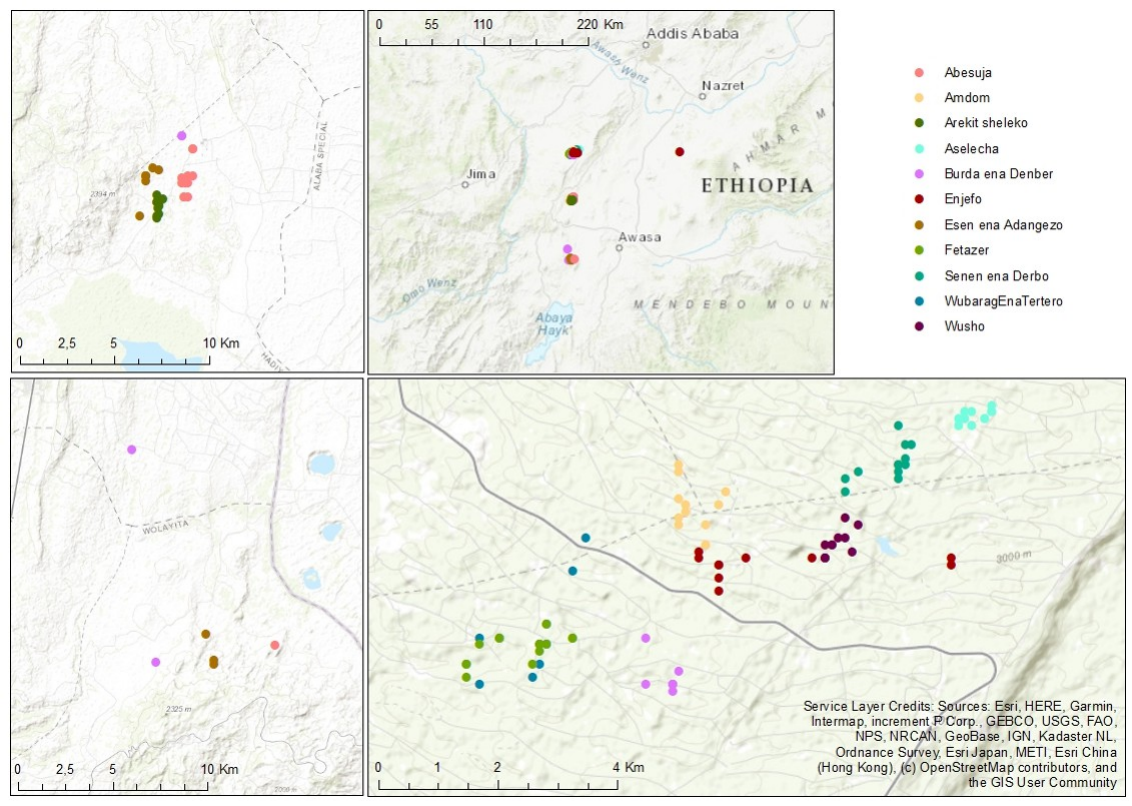
Map of the study area.

The Gumer highlands in the Gurage mountain range (Fig 1) rise out of the Ethiopian Rift Valley to elevations above 4,000 masl and form a chain roughly 100 km long (6° 49’ N 39° 47’ E). Major plants cultivated in the region include faba beans, peas, *Ensete* spp., potatoes, kale, garlic, and barley; with the latter being the major cash crop for farmers in this area. The annual rainfall pattern is bimodal, and mean annual temperatures range from 10°C to 21°C [19]. The study area map was developed in ArcGIS software using geographic coordinates data from a GARMIN 60 GPS.

The study area is ethnically and linguistically homogeneous, representing the Gurage people and language. The ethnic group has several clans, which are all active in managing society. There is an accessible asphalt road, but most farmers in the study area have little or no road access. Small-scale subsistence agriculture dominates the landscape. The smallest unit of the community is called a ‘kebele’ and comprises 100–500 farmers.

Eleven kebeles representing the highlands of Gumer were selected for interviews and collection of their barley landraces. The kebeles were selected based on their potential barley cultivation of landraces from the Gumer highlands. Eleven to 12 farmers were randomly selected from each kebeles for a total of 124 farmers (Table 1).

**Table 1:** Passport data for barley collection in Gumer.

No.	Landrace code	Common Name	Type	Use	Collector
**1**	GMP1	Chefe Awedo	Landrace	Food	Farmer
**2**	GMP2	Shemiya	Landrace	Food	Farmer
**3**	GMP3	Nech Senef	Landrace	Food	Farmer
**4**	GMP4	Awedo	Landrace	Food	Farmer
**5**	GMP5	Shehabdo	Landrace	Food	Farmer
**6**	GMP6	ChelkoTikur	Landrace	Food	Farmer
**7**	GMP7	JimuaTikur	Landrace	Food	Farmer
**8**	GMP8	Nech Temezhe	Landrace	Food	Farmer
**9**	GMP9	Keleme	Landrace	Food	Farmer
**10**	GMP10	Tikur Senef	Landrace	Food	Farmer
**11**	GMP11	Shege	Pure line selection from ‘1622–05’*	Food	Farmer
**12**	GMP12	Yebira Gebse (Traveler)	Released cultivar from HARC**	Malt	Farmer
**13**	GMP13	Jumua Nech	Landrace	Food	Farmer
**14**	GMP14	Jumua Tikur irregular	Landrace	Food	Researcher
**15**	GMP15	Chelko Tikur irregular	Landrace	Food	Researcher
**16**	GMP16	Senef irregular	Landrace	Food	Researcher
**17**	GMP17	Awedo irregular	Landrace	Food	Researcher
**18**	GMP18	Shemiya irregular	Landrace	Food	Researcher
**19**	GMP19	Jumua Nech irregular	Landrace	Food	Researcher
**20**	GMP20	Shahbdo irregular	Landrace	Food	Researcher
**21**	GMP21	Tikur Temezhe	Landrace	Food	Farmer
**22**	GMP22	Chefe awedo irregular	Landrace	Food	Researcher
**23**	GMP23	CO1***	Landrace	Food	Researcher
**24**	GMP24	CO2***	Landrace	Food	Researcher
**25**	GMP25	CO3***	Landrace	Food	Researcher
**26**	GMP26	CO4***	Landrace	Food	Researcher
**27**	GMP27	CO5***	Landrace	Food	Researcher
**28**	GMP28	CO6***	Landrace	Food	Researcher
**29**	GMP29	CO7***	Landrace	Food	Researcher
**30**	GMP30	Wesabo	Landrace	Food	Farmer

*: ‘1622–05’ is a landrace of the Ethiopian Biodiversity Institute.

**: HARC = Holeta Agriculture Research Center.

***CO = Code- given to the collected landraces which are identified by the researcher.

### Consideration of ethics and consent to participation

There is no rule for obtaining ethical approval for plant-related research projects in Ethiopia. To collect barley seed samples and interview farmers, a letter of support was obtained from the Hawassa University, Research and Technology Transfer Vice President Office, which was addressed to the Gumer district in the Gurage Zone, and a copy was provided to the administration of each of the eleven kebeles. The vice president of Hawassa University’s Research and Technology Transfer Office granted permission to interview and collect barley seeds from the farmer (S1 Text). Consent was obtained from the research participants who participated in the interview. The objective of the study was communicated to the participants. Participants were also assured that the information they provided would be kept confidential and would only be used for this study. Participants were asked about their gender, marital status, the size of their land, the number of children, education level, access to the market, access to improved seeds, the number of landraces on their farms, and names of landraces on their farms. Participants were also informed that they could withdraw from the study at any time during the data collection process if they felt the need to do so.

### Barley landrace collection and genotyping

A cross-sectional research design focused on selected kebeles within a district was identified after following a rapid preliminary informal survey and discussions with the zonal administration. Data were collected from farmers who cultivate barley landraces. Key information and personal observations were obtained through questionnaires, household interviews, and focus group discussions.

A questionnaire was used to collect information from a wide range of sources (respondents) on indigenous knowledge and practices involved in barley landraces and utilization in the study areas. The questionnaire was written in English and translated into the local language “Guragigna". The head of the family was chosen based on the preliminary survey and documents obtained from district agricultural offices.

Furthermore, all required age groups and sexes, including the heads of household of elder women, were purposefully included to ensure adequate coverage of the requirements. The mentioned landraces, with their names, use-value, and seeds, were collected during interviews. Each of the 124 barley sample communities was sampled during the main barley growing season in 2020. The total land planted with each crop was recorded for each farm. The SNP diversity was compared between barley from Gumer and collections from Japan, the US, and other locations in Ethiopia (S2 Table) that were chosen from a global barley diversity panel [24].

Individual barley samples (Table 1 and S1 Table) from each selected farm were characterized according to barley phenotypic characteristics. Seeds were collected from only one barley spike to ensure uniform barley genotype only one barley spike. Five individual seeds were allowed to germinate in Petri dishes for 4 days. The emerging primary leaf was collected for DNA extraction using a KCl extraction solution [25] at the Plant Cell Laboratory at Hawassa University, Ethiopia. The extracted DNA was preserved with 5 μl 95% ethanol and shipped to the Institute of Plant Science and Resources at Okayama University, Japan, for genotyping.

SNP genotyping was performed with the Illumina iSelect 50K barley SNP array [26]. Before sample genotyping, DNA quality was evaluated using a Nanodrop 2000 (Thermo Fisher Scientific, Waltham, MA, US) with the criterion for the 260/280 and 260/230 ratios to be more than 1.8. A Qubit (Thermo Fisher Scientific, Waltham, MA, USA) was used to quantify DNA, and 100 ng DNA per sample was used for scanning by the Illumina HiScan (Illumina, San Diego, CA, US). SNP alleles were identified using the Genome Studio Genotyping Module v2.0.2 (Illumina, San Diego, CA, US). SNPs with a minor allele frequency (MAF) of less than 5% and markers with more than 2 alleles were removed from further analysis. After quality checks, markers were assigned unique genomic locations, and their respective distances (in bp) were taken from the closest marker in the Morex reference genome. The reported end coordinates for each marker were recorded [27]. A total of 44,044 SNPs were detected. All monomorphic using default parameters, these markers were aligned to the Barley RefSeq v1.0 genome assembly [26] using default parameters. These were reduced to 20,368 biallelic loci after removing SNPs with more than 10% missing (S2 Table).

### Statistical analysis

To understand the diversity among the 11 kebeles, the Shannon-Wiener index, Pielou’s index, Pielou’s evenness, Simpson’s index, and abundance analysis were calculated using the vegan package (version 2.5–7) [28] with the following formulas:

$$Richness=\frac{\sum_{i}^{s}p_{i}}{s}$$

$$Menhinick’s\;index=\frac{\sum_{i}^{s}p_{i}}{\sqrt{s}}$$

$$Abundance=\frac{\sum_{i}^{s}p_{i}}{\sum_{i}^{s}S}$$

$$H’=\sum_{i=1}^{s}p_{i}\;{log}_{p}p_{i}$$

$$\lambda =\sum_{i=1}^{s}p_{i}^{2}$$

$$J’=\frac{H’}{\text{ln}(S)}$$

$$H=\frac{1}{\lambda S}$$

Where H is the Shannon index, *H’* is the Shannon-Wiener index, *J*’ is the Pielou index, pi is the proportion of barley landraces, S is the number of barley landraces and λ is Simpson’s index.

Genetic diversity was assessed for the Gumer collection and compared on the unbiased expected heterozygosity (He) from Ethiopian, Japanese, and USA populations chosen from the world barley panel. Genetic differentiation between populations was estimated with F statistics using the snpReady package (version 0.9.0) [29]. The snpReady is used for the preparation and quality control of datasets, the estimation of relationship matrices, and the estimation of basic population genetics parameters such as allele frequencies, minor allele frequency (MAF), observed heterozygosity (Ho), inbreeding coefficient (He), the effective population size, additive variance due to allele frequencies, variance in dominance due to allele frequencies, Nei’s genetic diversity, polymorphic information content (PIC), and Hardy Weinberg equilibrium (HW equilibrium) statistic. Linkage disequilibrium was calculated using the popper package (version 2.9.0) [30].

The spatial genetic structure of landraces was studied with principal component analysis (PCA) performed with the adegenet package (version 1.3–1) [31] using R statistical software (version 4.1.0) [32]. The population structure was also investigated using Structure 2.3.3 software [33, 34] with the admixture model and 25,000 burn-in iterations, followed by 50,000 Markov chain Monte Carlo (MCMC) iterations. The number of clusters (K) to describe the data was determined from the H value calculated by CLUMPP [35] and the ΔK value [34]. The results of 10 independent runs of Structure for each K value merged using CLUMPP, and the graphical output was obtained using the ggtree (version 2.1.3) [36] and the ggplot2 packages (version 3.3.3) [37].

## Results and discussion

### Farmers’ descriptions and identification of barley landraces

The common names assigned to genetic resources based on indigenous knowledge can be indicators of genetic distinctiveness (Table 1). Names are usually assigned based on distinctive characteristic features, specific or special end-use qualities, or other locally important attributes. As a result, evaluating and documenting such data are crucial parts of supporting conservation and future uses. We identified 30 different barley landraces in Ethiopia’s Gumer district (Table 1 and S1 Table). The landraces identified by the farmers have a local name that describes their characteristic values [38–42].

Seed color, spike length, number of rows, stress tolerance, yield, end-use characteristics, and other significant agronomic features distinguish the landraces. Some landraces are still being cultivated, with varying coverage in various locations. In contrast, Keleme is severely marginalized and on the point of extinction, while Shemeya has already vanished, as indicated by its presence only in one family. Similar findings have emerged from northern and Central Ethiopia, suggesting the rapid extinction of the major historical landrace.

The inter-landrace diversity or landrace richness (R), calculated using the Menhinick index, is summarized in Table 2. All indices indicated an exceptional landrace richness in all kebeles studied (Table 2). The farmers in the region grew their landraces on their farms (Tables 2 and 3), although the amount of on-farm diversity differed across kebeles. Farmers in the Aselecha and Wusho kebeles grew more than three landraces of barley; the Aselecha kebele had the highest average number of barley on-farm (3.58), followed by the Wusho kebele (3.27). All diversity indices measured were not significantly different across the sampled kebeles (*P*> 0.05, Table 2). The implication is that all kebeles adopt comparable barley landraces. The relative abundances of barley samples showed that communities tended to evolve towards a similar structure of barley landraces, where 2 to 3 types of barley landraces are planted in their farming fields every year.

**Table 2:** Summary of diversity statistics for the barley landraces collected in Gumer.

Name of kebele	Average no. of improved cultivars	Average no. of landraces	Richness (%)	Menhinick’s index	Abundance	Shannon index (H)	Simpson’s index (λ)	Pilou eve ness (J)	Shannon-Wiener index (H’)	Altitude (m) Min	Altitude (m) Max
**Abesuja**	1	2.91	46.7	1.73	32	1.79	0.82	0.42	5.97	2,730	2,849
**Amdom**	0.9	3.17	53.3	1.92	38	1.93	0.84	0.4	6.9	2,840	2,965
**Arekitshleko**	0.9	2.73	46.7	1.76	30	1.77	0.81	0.42	5.9	2,829	2,901
**Aselecha**	1.6	3.58	66.7	2.38	44	2	0.84	0.36	7.37	3,150	3,265
**Burdaenadenber**	1.1	2.91	60	2.31	32	1.97	0.84	0.38	7.16	2,901	2,967
**Enjefo**	1.1	3.18	60	2.25	35	1.95	0.83	0.38	7.04	2,934	3,021
**Esenenaadangezo**	1.1	3.09	46.7	1.99	34	1.88	0.82	0.4	6.54	2,831	2,925
**Fetazer**	1.1	2.64	46.7	1.48	29	1.68	0.8	0.44	5.39	2,821	2,908
**Senenenaderebo**	0.9	3.08	60	2.22	37	1.91	0.83	0.38	6.74	3,018	3,153
**Wulbaragenatereteo**	1.1	2.73	46.7	2.04	31	1.78	0.79	0.38	5.93	2,813	2,909
**Wusho**	1	3.27	46.7	1.4	36	1.74	0.82	0.46	5.71	2,829	2,931

*The average number of improved cultivars and landraces is calculated based on the number of farmers who owned the landrace in each kebele.

**Table 3:** Summary of characteristics of barley genotype identified by the surveyed household.

*No*	Local name	Caryopsis type	Seed color	Row No.	Preferred characteristics	Non-preferred characteristics
***1***	Chifeye -awedo	Covered	White	Two	Seed color, best food quality, medicinal value, and best for roof thatching	Intermediate yield
***2***	Shemeya	Covered	Purple	Two	Medicinal value, disease and drought tolerant and, best for roof thatching	Late maturing, low yield, and less market demand
***3***	Nech-senef,	Covered	Yellow	Two	Long, high market price, best for ‘kolo’ and needs less labor for dehulling	Requires fertile soil, low yield, and susceptible
***4***	Awedo	Covered	White	Two	Seed color, best food quality, and best for roof thatching	Intermediate yield
***5***	Sheh abdo	Covered	White	Six	High yield, long height, and spike	Less market demand and less food quality
***6***	Chelko-tikur	Covered	Grey	Six	Early maturing, drought and disease-tolerant, high yield, long spike, and roof thatching	Shattering before harvest and low market price
***7***	Jimua tikur	Covered	Black	Six	High yields that tolerate early maturing, drought and disease-tolerant high yield, and for roof thatching	Low market price
***8***	Nech-temezhe	Naked	White	Two	No need for labor for dehulling and early maturing	Low yield difficulty for threshing, and less food quality
***9***	Keleme	Covered	White	Six	Sweet taste, intermediate yield, and requires less labor for dehulling	Susceptible and less market demand
***10***	Tikur-senef	Covered	Black	Two	Long, requires less labor for dehulling and is best for ‘kolo’	Less market demand, susceptible, requires fertile soil and low yield
***11***	Shege	Covered	White	Six	Long, high yield in the first year and high market price	Seed cost per year, and less food quality
***12***	Yebira-gebs	Covered	White	Two	High yield and high market price	Short plant height and seed cost
***13***	Jimua nech	Covered	White	Six	Early maturing, high yield, and roof thatching	Less market demand and less food quality
***14***	Tikur Temezhe	Naked	Black	Six	No need for labor for dehulling and early maturing	Low yield, difficulty in threshing, and less food quality
***15***	Wesabo	Covered	Variegated	Six	Drought and disease-tolerant, high yield, roof thatching	Less market demand due to its seed color

We also found that the diversity estimate (Pilou evenness (J)) was high, except for the Aselecha kebele, which scored 0.36 (Table 2). The cultivation of different landraces and, therefore, the high abundance of the majority of the landraces identified in the kebeles led to the highest unevenness in the districts (the lowest admixture structure). Our research suggests that this study area has the potential for barley breeding and conservation.

Farmers in the study districts intentionally preserve landraces to meet their needs (Table 3). These requirements include, but were not limited to, early or late planting (i.e., maturity), yield potential with the type of environment intended to be grown, soil conditions (i.e., waterlogged, fertility, or frost effects), and intended dishes and beverages (includes quantitative and qualitative aspects such as product volume, taste, visual appeal, color, and storage ability). Thirty landraces with varying maturity, yield potential, stress tolerance, end-use characteristics, and other agronomic traits were grown. Table 3 summarizes the user preference of some of the landraces cultivated by farmers, which provides insight into the diversity of landrace types and their management as described by farmers. The landraces’ names and descriptions reflect key quantitative, qualitative, and end-use characteristics, planting time, or origins. Farmers listed the following landraces as the most common: Chifeyeawedo, Shemeya, Nech-senef, Awedo, Sheh abdo, Chelko-tikur, Jimua tikur, Nech-temezhe, Keleme, Tikur-senef, Jimua nech, and Wesabo. The landraces are six and two-rowed. Hulled and hulless; black, and white.

Gender plays a role in selecting which landrace to plant. Women favor Keleme for its sweet taste, easy dehulling, and medicinal value. But men do not prefer it due to its susceptibility to drought, disease, and low market value (Table 3). All farmers select Awedo for its white color, suitability for roof thatching, and market value (Table 3).

Farmers cultivate a diverse range of landraces, each with its maturity, yield potential, stress tolerance, end-use quality, and other agronomic characteristics [43]. This discovery is similar to how other Ethiopian farmers preserved many barley landraces for future use [44]. Rice landraces in the Himalayas are preserved for different purposes [45]. Similarly, farmers in Peru grow various types of potatoes to resist blight [46]. This custom is also similar to the culture of maize in Mexico [47], where colored maize is highly preferred. Similar practices exist in the case of tetraploid wheat in central Ethiopia for its aroma and taste preference [48]. A comparable trend exists in Tibet, where landraces are preserved for cultural value [49].

Fifteen land races planted in the study areas are identified, named, and described by Gumer farmers. Since SNP markers differentiated the landraces, vernacular names and farmer descriptions of landraces could be linked to formal scientific classifications. The landraces have been identified as a distinct type using the scientific taxonomic classification system using morphological traits [50].

The selection and decision-making process was based on sets of criteria. This value has an impact on the successful management of the landraces for their cultural and environmental needs. Complexity is expressed in their description of landraces for adaptation, stress tolerance, and yield (Table 3). Selection criteria often reflect changes in farming conditions and responses to socioeconomic and cultural factors that influence farmers’ priorities [51]. Factors that influence barley production and diversity are end-use, biotic, and abiotic tolerance of the landraces.

### Genetic diversity of barley

In the present Gumer barley panel, we identified 20,367 replicable SNP calls of the 44,040 SNP probes available on the array (S2 Table). Of these, 40716, 32216, 40292, and 40631 alleles were found among barley collections from Ethiopia, Gumer, Japan, and the USA, respectively. The numbers of polymorphic SNP markers examined were 91, 872, 4,897, 4,134, 3,573, 5,190, 3,141, and 3,819 on chromosomes 0H, 1H, 2H, 3H, 4H, 5H, 6H, 7H, respectively. The S3 Table shows the observed number of heterozygotes (Ho), the expected number of heterozygotes under panmixia (He), and the coefficient of inbreeding (Fis) for each of the 20,367 SNPs. The ratio of the expected minimum, maximum, and observed expected number of heterozygotes was 0.08, 0.51, and 0.30, respectively. The expected and observed heterozygosity among the SNPs was 0.30 and 0.03, respectively. Positive multilocus estimates of FIS (0.9) have been shown. The value for the Bartlett test of homogeneity of variances between observed and expected heterozygosity showed Bartlett’s K-squared = 2157.4, df = 1, p-value < 2.2e-16 indicating the presence of a significant difference between expected and observed heterozygosity.

We compared the Gumer barley SNP markers to Japanese, USA, and other Ethiopian landraces. The PIC value range was 0.17 to 0.38, with an average value of 0.30. Nei’s gene diversity score ranged from 0.15 to 0.50, with a mean of 0.38. The MAF ranges from 0.10 to 0.50 with an average value of 0.28, indicating the presence of a great diversity among the barley landraces (Table 4).

**Table 4:** Ranges of Nei’s gene diversity (GD), polymorphic information content (PIC), minor allele frequency (MAF), observed heterozygosity (H_o_), inbreeding coefficient (Fi), and effective alleles (N_e_), number of landraces (N), number of markers (# SNPs), the index of associations (IA), and linkage disequilibrium testing (rbarD), *P*-values <0.01) of the 20,3670 polymorphic SNPs generated from collections from Gumer, Ethiopia, Japan, and the USA.

Collection places	Parameters	GD	PIC	MAF	H_o_	F_i_	N_e_	N	#SNPs	*IA* and rbarD	*p-value*
**Overall**	Mean	0.38	0.3	0.28	0.03	0.93	98.1	182	20,369		
	Lower	0.18	0.17	0.1	0.01	0.15					
	Upper	0.5	0.38	0.5	0.32	0.97					
**Gumer**	Mean	0.31	0.25	0.21	0.15	0.51	29.5	30	4,259	2776.86 (0.26)	0.01
	Lower	0.18	0.16	0.1	0.05	-0.28					
	Upper	0.5	0.38	0.5	0.4	0.83					
**Ethiopia**	Mean	0.37	0.3	0.27	0.03	0.92	29.4	54	19,875	1078.05 (0.05)	0.01
	Lower	0.18	0.17	0.1	0.01	0.38					
	Upper	0.5	0.38	0.5	0.23	0.96					
**Japan**	Mean	0.37	0.3	0.28	0.03	0.91	15.9	29	18,815	1363.93 (0.07)	0.01
	Lower	0.19	0.17	0.1	0.01	0.14					
	Upper	0.5	0.38	0.5	0.32	0.96					
**USA**	Mean	0.39	0.31	0.29	0.02	0.94	36.5	69	20,233	341.20 (0.02)	0.01
	Lower	0.18	0.17	0.1	0.01	0.73					
	Upper	0.5	0.38	0.5	0.1	0.97					

The PIC values were 0.25, 0.30, 0.30, and 0.30 for Gumer, Ethiopia, Japan, and the US, respectively. The average gene diversity (GD) was 0.31, 0.37, 0.37, and 0.39 for Gumer, Ethiopia, Japan, and the United States, respectively (Table 4, S3 Table). Similarly, there was a difference in the average MAF of the four geographic locations, which was 0.21 (GUM), 0.27(ETH), 0.28 (Japan), and 0.29 (USA). These results showed that there was high genetic variation among the 182 barley landraces. GD (0.39) was the highest for barley from the USA compared to Gumer (Table 4). In contrast, landraces from the experimental area had the lowest PIC and GD scores. The average Ho in the 30 landraces of Gumer farmers (0.15) was significantly higher than in the collections of Japan (0.03), the United States (0.02), and Ethiopia (0.03). Finally, the inbreeding coefficient (FIS, 0.51) in Gumer barley was significantly lower than in Ethiopia (0.92), Japan (0.91), and the USA (0.94) populations.

The genetically effective size of populations was determined using SNP data (Table 4). The Japan collection showed the lowest Ne value (15.9), while the USA collection showed the highest Ne value (36.5). The Gumer collection showed the greatest ratio of Ne/No. of genotypes (29.5/30 = 0.98) between collections. The result indicates that inbreeding and genetic drift among the Gumer landraces were higher than the samples from other populations. The result explains the farming practices of Gumer, in which local landraces are grown with few improved varieties. This high self-reflection rate causes considerable distortion from Hardy-Weinberg equilibrium (HW), which shows only 249 loci in HW equilibrium (S3 Table). We also observed that 30, 54, 29, and 69 individuals from Gumer, Ethiopia, Japan, and the United States had P = 0.017, 0.01, 0.01, and 0.01 for the average association index (r d) = 0.256, 0.05, 0.07, and 0.02, respectively (Table 4). There is strong evidence of linkage disequilibrium between loci based on the association index, IA (P = 0.02), which is consistent with a population of self-pollinating and inbreeding. The other populations (Ethiopia, Japan, and the USA) clustered into two and more than two with a little too high admixture. The hypothesis of no marker linkage was rejected (*P* < 0.001), indicating a population of self-pollinating and inbreed in Gumer.

### Population structures of landraces

**Fig 2:**
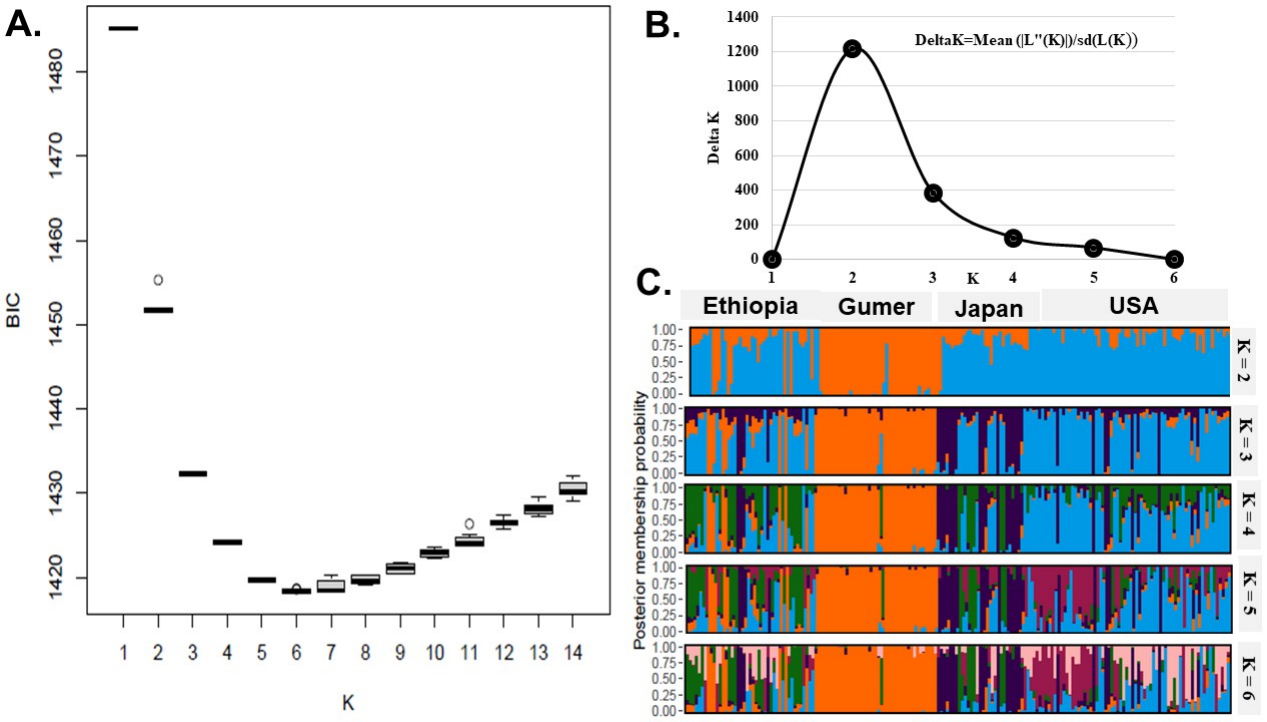
Distribution and population structure of barley landraces in the Gumer district compared to the landraces of barley from Ethiopia, Japan, and the USA barley landraces. The colors indicate the geographic origins of the barley samples. A. Bayesian information criterion (BIC) for a range of K values (lower BIC indicates higher model support). B. Estimation number of populations using the delta K derived from LnP (D) from 1 to 6 using SNP data. C. Assignment plots where each vertical bar represents an individual. The y-axis is posterior membership probability, and the x-axis is individuals within their population.

We determined the most likely population structure for the landraces using two methods. The first technique was a genetic structure-based clustering strategy, which was inferred using the second-order rate of change of the likelihood K (Fig 2B and S4 Table). We found a distinct peak at K = 2, and the delta K value was 1,200, indicating that the populations in the study were best classified into two groups. The second method was based on discriminant principal components (DAPC) analysis). This method should show an elbow at a specific K value, where the Bayesian information criterion (BIC) would be the lowest [52]. This pattern was observed in our data. BIC values decreased up to K = 6 and increased from K = 7 to K = 14 (Fig 2A). This situation suggested that fewer than six clusters could be ideal. As a result of the BIC inferred clustering result, we grouped the landraces into six clusters (P1–P6). The six populations contained 51, 29, 28, 68, 33, and 3 landraces, respectively (Fig 2).

We found that Bayesian model-based stratification and PCA clustering techniques did not group barley collections based on their geographic origin. For example, a variety of Gumer (GM12) clustered along the USA. A higher number of polymorphic SNPs were found in the United States and Japan landraces compared to those in Gumer.

**Fig 3:**
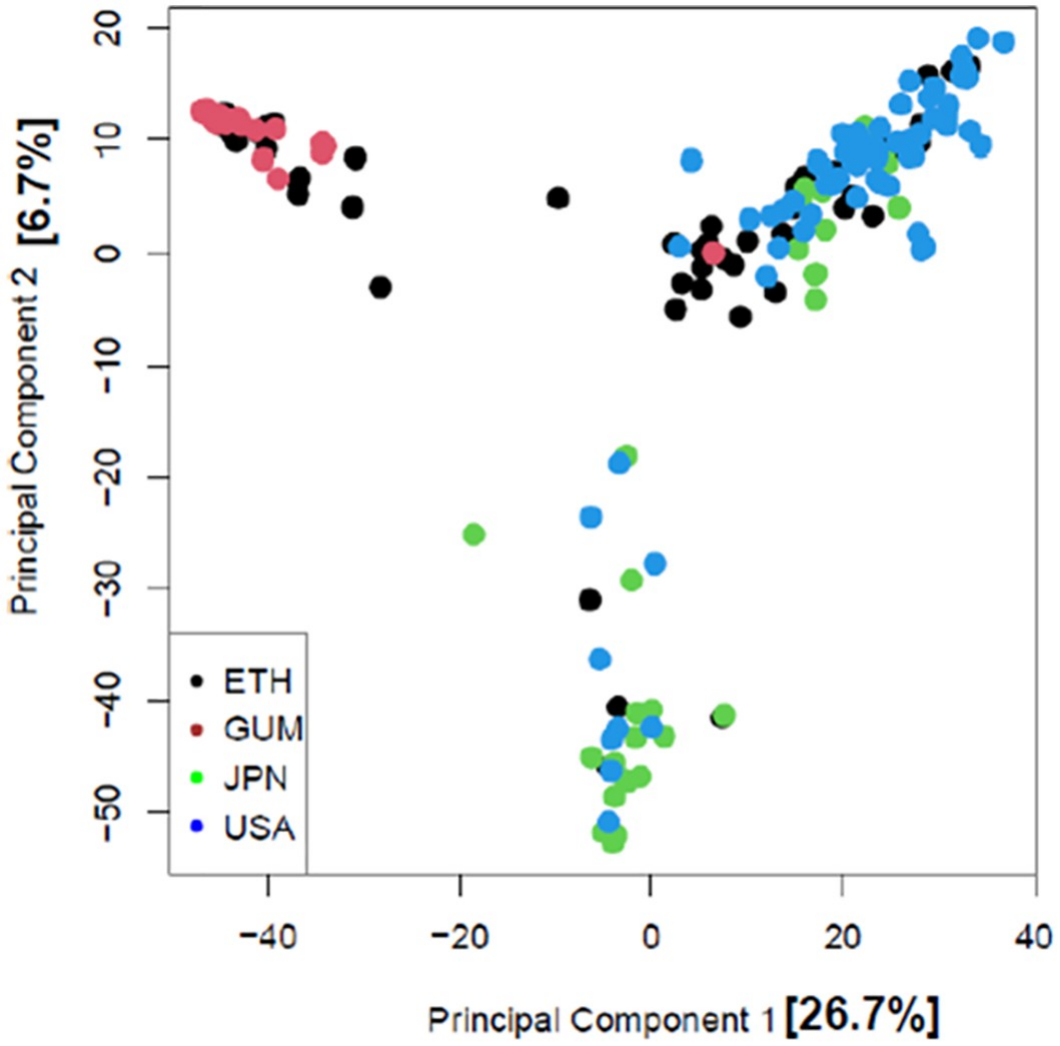
Scatter plot of the first two components of the principal component analysis of Gumer, Ethiopia, Japan, and US barley. The color of the dot indicates the geographic origin.

A PCA analysis revealed that location on a continental scale is not the most predictor of genetic structure. The geographic origin of the landraces did not confirm the clustering in the first components (PC I), which together explain 26.7% of the variance. PC1 distinguishes Gumer and some Ethiopian barley from the US barley, while PC2 distinguishes the USA and Japan barley from Gumer and some Ethiopian barley (Fig 3).

**Fig 4:**
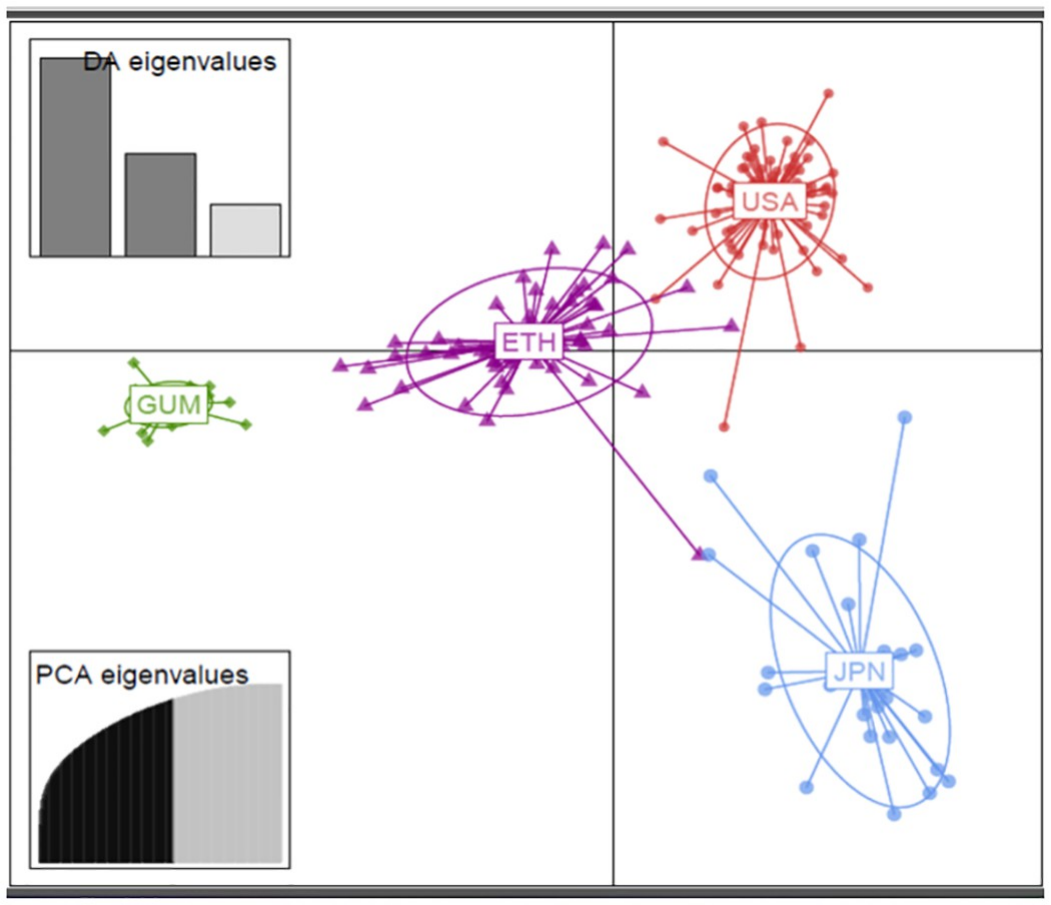
Discriminant analysis of principal components (DAPC) for 182 barley genotypes from USA, Japan, Ethiopia, and Gumer. The first two linear discriminants are represented by the axes (LD). The scatterplot of the final DAPC model with sampling locality as a prior, where points are individual genotypes, color-coded by their original sampling locality, and surrounded by a 95% confidence ellipse. The DA and PCA eigenvalues represent the amount of genetic variation captured by the analysis, and the first two discriminant factors are plotted as the x- and y-axis. Clusters 1, 2, 3, and 4 represent Gumer, Ethiopia, Japan, and USA collections.

DAPC analysis (Figs 2 and 4) reveals differences between groups as clearly as possible. The lowest BIC value (1,418) was found to correspond to K = 6 (Fig 2A). Hundred PCA axes and all discriminant functions were kept for DAPC analysis. There were six clusters between 182 barley landraces compared in this study on the scatterplot of two main components of DAPC (Figs 2 and 4).

The clusters were composed of Gumer, Ethiopia, Japan, and USA samples. Populations were divided according to their collection origin; thus, continuous genetic flow between populations is limited or absent (in the case of Gumer). This was confirmed by the unique structure in the Gumer population, shown by the Structure, DAPC, and PCA analysis. Simultaneously, the use of PCA, Structure, and DAPC in clustering the barley landraces from different geographic regions yielded comparable results, indicating a strong genetic uniqueness of Gumer barley compared to USA and Japan barley (Figs 2–4). The study’s use of these methodologies showed a unique structure of Gumer barley attributable to the farming community’s history of barley selection in the region.

### Comparison of variation among landrace groups

The results of AMOVA revealed that the variation accounted for 71%, 50%, and 58% of total genetic variability for Ethiopia, Japan, and the United States, respectively (Table 5). By contrast, AMOVA among the four populations was 75%. Genetic divergence was 19% (Gumer), 41% (Ethiopia), 36% (Japan), and 18% (overall selected population).

**Table 5:** Analysis of molecular variance by collection region.

Comparison of population	Source	Degree of freedom	MS	Estimated variance	Sigma	% of total variation	Fixation index (F_ST_)	Gene flow (N_m_)
**All population**	Between pop	3.0	7399.0	2466.3***	27.5	41.0	0.41	0.36
	Between samples Within pop	178.0	13658.4	76.7***	37.2	55.5	0.94	
	Within samples	182.0	427.0	2.3***	2.3	3.5		
	Total	363.0	21484.4	59.2***	67.1	100.0	0.97	0.01
**Gumer vs USA**	Between pop	1.0	6586.9	6586.9***	78.1	74.3	0.74	0.09
	Between samples Within pop	97.0	5074.7	52.3***	25.2	24.0	0.93	
	Within samples	99.0	182.0	1.8***	1.8	1.7		
	Total	197.0	11843.7	60.1***	105.2	100.0	0.98	0.00
**Gumer vs. Ethiopia**	Between pop	1.0	2355.5	2355.5***	29.4	39.8	0.40	0.38
	Between samples Within pop	82.0	7126.0	86.9***	42.4	57.4	0.95	
	Within samples	84.0	177.0	2.1***	2.1	2.9		
	Total	167.0	9658.5	57.8***	73.9	100.0	0.97	0.01
**Gumer vs Japan**	Between pop	1.0	3427.3	3427.3***	57.3	68.3	0.68	0.12
	Between samples Within pop	57.0	2859.6	50.2***	23.6	28.2	0.89	
	Within samples	59.0	174.0	2.9***	2.9	3.5		
	Total	117.0	6460.8	55.2***	83.8	100.0	0.96	0.01
**Ethiopia vs. Japan**	Between pop	1.0	619.9	619.9***	6.8	11.1	0.11	2.00
	Between samples Within pop	81.0	8583.7	106.0***	51.5	84.1	0.95	
	Within samples	83.0	245.0	3.0***	3.0	4.8		
	Total	165.0	9448.6	57.3***	61.3	100.0	0.95	0.01
**Ethiopia vs USA**	Between pop	1.0	1494.1	1494.1***	11.6	20.3	0.20	0.98
	Between samples Within pop	121.0	10798.8	89.2***	43.6	76.2	0.95	0.01
	Within samples	123.0	253.0	2.1***	2.1	3.6		
	Total	245.0	12545.9	51.2***	57.2	100.0	0.96	0.01
**Japan vs. USA**	Between pop	1.00	604.28	604.28***	6.57	15.68	0.16	1.34
	Between samples Within pop	96.00	6532.39	68.05***	32.75	78.22	0.94	0.02
	Within samples	98.00	250.00	2.55***	2.55	6.09		
	Total	195.00	7386.67	37.88***	41.86	100.00	0.93	0.02

The sigma reflects the total variance explained by each source and for each population. The Phi value indicates the population differentiation statistics. A higher Phi number indicates a higher degree of differentiation. The values of Sigma and Phi are statistically significant at *P* < 0.001 (***).

Based on SNP markers, the overall pairwise F_ST_ value was 0.41 (*P* 0.001). However, the value of F_ST_ between Gumer and Ethiopia, Gumer and Japan, Gumer and USA, Ethiopia and USA, and Japan and USA was 0.40, 0.68, 0.74, 0.11, 0.2, and 0.16, respectively (Table 4).

AMOVA showed a clear structure between the collection sites (Gumer vs. Ethiopia) and the geographic origins (Japan, USA) in this study. These findings indicate the need for a specific collection site and use of the value of barley to unravel the different human practices in domesticating barley for different use values. When comparing Gumer vs. Ethiopia, FST was 0.40, and Nm was 0.38. Nm between Japan and USA was high, and genetic differentiation based on FST values was significant between the landraces among the different populations, which confirmed the dynamic genetic variation among the collected landraces [53, 54]. The findings also confirm the presence of significant diversity in Ethiopia and Gumer barley landraces. The results could be best explained by historically effective population sizes [55], while genetic differentiation is influenced by recent changes caused by the use of barley for the local population [56].

**Fig 5:**
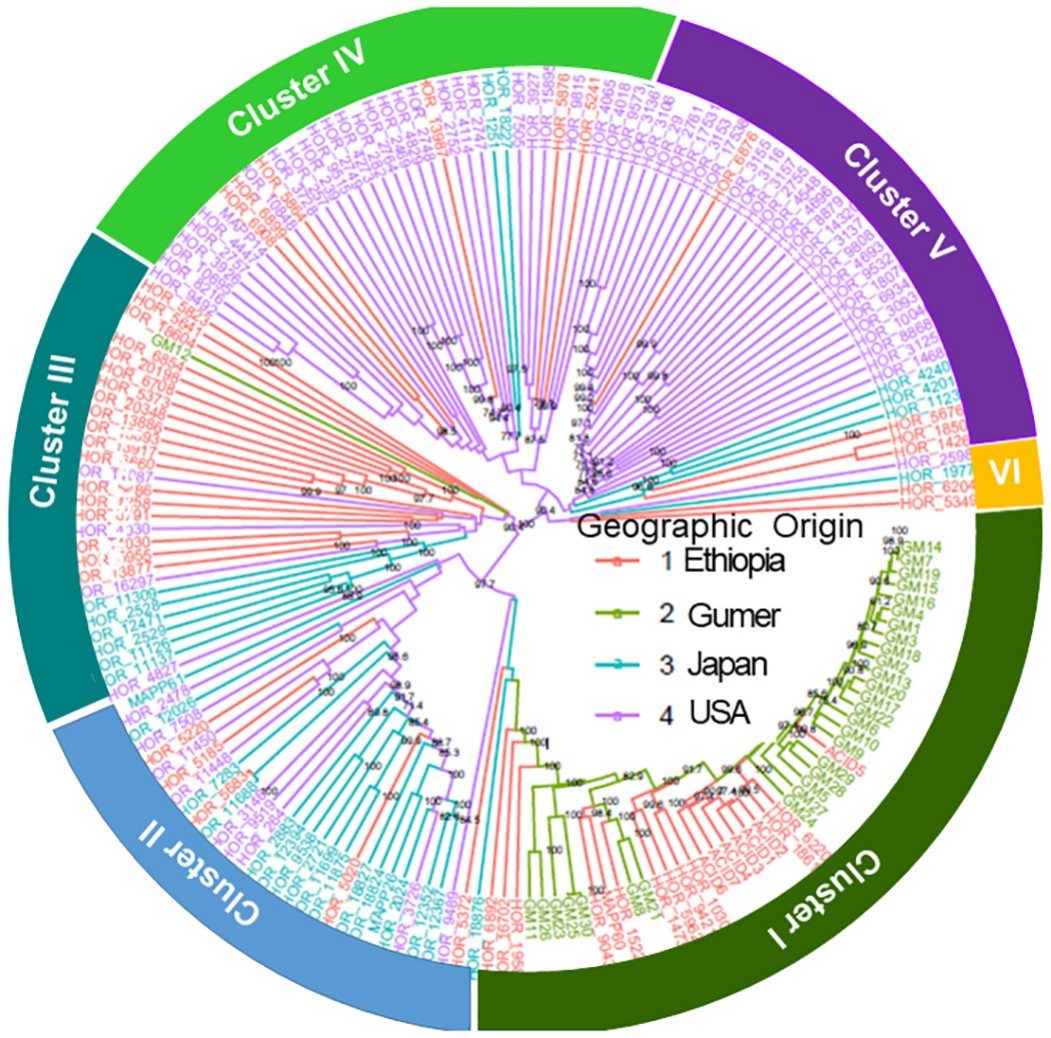
Maximum parsimony tree. Tree based on a binary matrix calculated from a dataset of 20,367 SNPs analyzed in 182 barley landraces from four different geographic regions. Clustering was supported by bootstrap analysis with 1000 replicates. Next to strain numbers, different colors represent the places from which the barley landraces were collected. Different circular curves indicate clusters of the landraces.

The clustering analysis revealed that the SNP data did not classify landraces based on their geographical background, and landraces were jumbled together. For example, cluster I comprised 29 Gumer landraces and 18 landraces from Ethiopia, a landrace from the USA, and Japan. Cluster II comprised 17 landraces from Japan and 10 landraces from the USA, and 4 landraces from Ethiopia. Cluster III included 6 Japanese, 4 American, 1 Gumer, and 18 Ethiopian landraces. Cluster IV contained landraces from the United States, with only six Ethiopian and two Japanese landraces. Cluster V was made up of landraces from the United States, with three from Japan and four from Ethiopia. Cluster VI is composed of 1, and 2 Japanese and Ethiopian landraces (Fig 5).

We discovered that the Ethiopian landraces were closer to the Japanese landraces and far from the Gumer landraces, rather than the two groups being closer to each other, using Fst values between populations (Table 6). The Japan and USA landraces were closer to each other than the Gumer landraces (Table 6), which was also supported by the hierarchical clustering of genetic distance, which revealed that the Gumer landraces form a distinct patch (Fig 5).

**Table 6:** Genetic distance between populations using Fst.

		**P-values**			
		ETH	GUM	JPN	USA
**F**_**ST**_	ETH		0.00	0.00	0.00
	GUM	0.40		0.00	0.00
	JPN	0.11	0.68		0.00
	USA	0.20	0.74	0.16	

ETH (Ethiopia), GUM (Gumer), JPN (Japan), and USA (United States of America) FST is the population differential due to genetic structure, while P-values are the probability of accepting and rejecting the FST result.

Some Ethiopian landraces clustered with Japanese and US landraces, which implies that they share a genetic background (Fig 5). This grouping result is remarkably similar to the previous one. Similar to the findings of a large-scale genotyping study of barley germplasm [57]. Ethiopia does not share borders with Japan, and the USA, thus plant and animal species will not frequently cross those borders, so the clustering of the Ethiopian landrace along with the Japan and USA landrace can only be explained by the presence of germplasm exchange through breeding [57]. This grouping of landraces is also in the STRUCTURE analysis, where the Gumer population has a unique genetic structure. These findings should be validated with a large set of germplasm.

### Genetic diversity of landraces in Gumer

Genetic diversity is essential to create a robust food security system capable of responding to repeated biotic and abiotic stresses [58]. In plant breeding, genetic diversity analysis is critical in identifying alleles to develop crops with high yield, resistance to biotic and abiotic stressors, and satisfactory production [53].

Our interviews with the farmers showed that most landraces are used for food. These findings provide insight into the observed patterns in the Gumer barley genetic structure. We found little difference in abundance among the kebeles’ collections, likely due to the exchange of landraces among the members in different Kebele. The community uses barley as a food source, animal feed, and a source of income. As a result, the farmers select landraces with higher yields due to their increased adaptation to local farming conditions. These practices, and the importance of barley to the Gumer communities, shed light on the low diversity and genetic structure of barley in Gumer.

The 50K barley SNP array is designed to capture the most reliable gene-associated SNP markers available in the barley genome (cv. Morex), but it is not sufficient to discover novel loci from Gumer landraces. The fact that we found fewer replicable SNPs on the array than the original Morex allele panel suggests the presence of new alleles. Subsequent research would benefit from hybridization and sequencing techniques to offer a more comprehensive description of the Ethiopian barley genome.

Our findings demonstrated that the barley landraces gathered from Gumer had lower diversity indices than those gathered from other regions in Ethiopia. This suggests that the occurrence of variability between the Ethiopian and Gumer landraces may have been caused by the usage of farmer landraces that are stored and employed for every season of farming. This study’s genetic diversity and structure could be attributable to genetic drift that occurred during the rejuvenation cycles. Several conditions must be met for this to be a persuasive explanation. For instance, the initial samples used to create the early landraces must have been large enough to capture a significant amount of the genetic diversity prevalent in today’s landrace populations in Gumer. In addition, the genetic diversity of *in situ* landrace populations must not have changed systematically over time. Alternatively, the genetic diversity in Gumer landraces could arise from factors such as adaptation to wider agroecology, natural crossings caused by cultivating different genotypes in a field, and the use of diversified agricultural methods.

We identified two barley subpopulations using a genetic stratification approach based on a Bayesian clustering model. A discriminant analysis using the BIC suggested that fewer than six clusters would be preferable. Both methods showed successful clustering of Gumer barely separate from USA, Japan, and other Ethiopia landraces.

Another group used barley from gene banks and found [59, 60] geographic origin as a primary region or structure. However, our results show different facts where the structure of barley is not based on geographic location. For example, Ethiopian landraces have a structure similar to USA and Japan. And some Ethiopian landraces have the same structure as the Gumer landraces. The presence of multiple groups of Ethiopian landraces could be due to frequent germplasm exchanges between different countries or to the fact that humans manipulated barley genes throughout history, resulting in genetic mixing through breeding.

However, our study found that there was limited admixture between the current barley collection from Gumer and barley from Japan, the USA, and other regions of Ethiopia. Instead, Gumer barley formed a separate structure and cluster, at least from Japan, and USA landraces, demonstrating a unique genetic makeup; therefore, the landraces now represent a unique genome structure of barley.

## Conclusion

We collected 30 barley landraces from Gumer in this work and compared them to landraces from Ethiopia, Japan, and the USA, using a high-density 50K barley SNP array. The research assesses the genetic diversity and population structure within landraces. Our results revealed a moderate polymorphism in the investigated Gumer barely. The molecular analysis in the tested landraces from Ethiopia, Japan, and the USA showed a high level of SNP variability. Based on the results of AMOVA, genetic diversity observed within Gumer is less than that found among samples from Ethiopia, Japan, and the USA. The result suggests that local landraces have less genetic differentiation. In addition, based on obtained results, the SNP array can distinguish the Gumer barley from Ethiopia, Japan, and the USA. However, many SNP lines were not recognized from the Gumer genome, indicating that a reference genome for Ethiopian barley germplasm is an alternative method to unravel the domestication process of Ethiopian barley. This study highlights the value of farmer-based agroecology in efforts to quantify diversity in barley. As a result, DNA genotyping of barley collection using a large number of landraces and SNP markers tightly linked to known genes associated with plant adaptation to different environments can help breeding programs based on the deployment of specifically selected DNA markers spread across the entire genome. The creation of sets for these chosen SNP markers of adaptation-related genes and their use in breeding programs could speed up the production of new competitive barley cultivars.

## Supporting information

S1 Table Passport data of the Gumer barley landraces. (XLSX)

S2 Table Highly informative selected single-nucleotide polymorphism markers for genetic diversity analysis, and data for calculating barley diversity among the different Kebeles of Gumer. (XLSX)

S3 Table Genetic diversity in the 182 landraces of barley. Summary statistics of genetic variation existing in barley based on 20368 SNP among 182 landraces. H_o_: heterozygosity within a population; H_s_: genetic diversity within a population; H_t_: overall gene diversity; H_ttp_: corrected H_t;_ D_st_: gene diversity among samples; D_stp_: corrected D_st_; F_st_: fixation index; F_stp_: corrected F_st_; F_is_: inbreeding coefficient per overall loci; D_est_: a measure of population differentiation. Significant deviation from Hardy–Weinberg equilibrium at *P < 0.05, **P < 0.01, and ***P < 0.001, respectively.
(XLSX)

S4 Table Determining the structure barley landraces using a structure harvester. The results are based on allele frequencies for 20368 SNP markers in 182 barley landraces.
(DOCX)

S1 Text Support letter confirming the authorized institute that permits barley collection and farmer interviews. (PDF)
