# Supplementary material for: Genetic diversity and population structure of barley landraces from Southern Ethiopia’s Gumer district: Utilization for breeding and conservation
Source: PLoS One. 2023 Jan 5;18(1):e0279737. doi: 10.1371/journal.pone.0279737 (PMC9815628; doi:10.1371/journal.pone.0279737)
Supplement: S4 Table — The results are based on allele frequencies for 20368 SNP markers in 182 barley landraces. (DOCX) [file pone.0279737.s004.docx]

| K | Mean LnP(K) | Stdev LnP(K) | Ln'(K) | Ln''(K) | DeltaK |
| --- | --- | --- | --- | --- | --- |
| 1 | -4187828.62 | 31.23 | NA | NA | NA |
| 2 | -3449603.1 | 325.05 | 738225.52 | 395545 | 1216.87 |
| 3 | -3106922.18 | 456.36 | 342680.92 | 175902 | 385.45 |
| 4 | -2940143.06 | 466 | 166779.12 | 58631 | 125.82 |
| 5 | -2831994.98 | 333.51 | 108148.08 | 22595.6 | 67.75 |
| 6 | -2746442.54 | 285.58 | 85552.44 | NA | NA |
|  |  |  |  |  |  |

S4 Table. Determining the structure barley landraces using a structure harvester. The results are based on allele frequencies for 20368 SNP markers in 182 barley landraces. LnP (D) and ΔK, were calculated according to [1]. Data is plotted in Fig. 2B

1. Earl DA, vonHoldt BM. STRUCTURE HARVESTER: a website and program for visualizing STRUCTURE output and implementing the Evanno method. Conserv Genet Resour 2011 42. 2011;4: 359–361. doi:10.1007/S12686-011-9548-7
