# Supplementary material for: Genetic diversity and population structure of barley landraces from Southern Ethiopia’s Gumer district: Utilization for breeding and conservation
Source: PLoS One. 2023 Jan 5;18(1):e0279737. doi: 10.1371/journal.pone.0279737 (PMC9815628; doi:10.1371/journal.pone.0279737)
Supplement: S1 Text — (PDF) [file pone.0279737.s005.pdf]

ሀዋሳ ዩኒቨርሲቲ
የምርምርና ቴክኖሎጂ ሽግግር
ም/ፕሬዚዳንት ጽ/ቤት

Hawassa University
Research and Technology Transfer
Vice President Office

ቁጥር
Ref. No. VPRTT/087/21
ቀን
Date: 26/10/2021

Dragan Perovic, Ph.D

Academic Editor, Journal of PLOS ONE

Re Support letter

Dear Editorial Board,

The purpose of this letter is to inform you that Hawassa University Research Office have granted Hewan Demissie Degu (PhD) permission to conduct research at College of Agriculture, School of Plant and Horticulture Science, titled "Genetic Diversity and Population Structure of Barley Landraces from Southern Ethiopia's Gumer District: Utilization for Breeding and Conservation."

This also ensures that this school adheres to the criteria of the right to gather barley from farmers' land and the protection of farmer's landrace. We want to make sure that all of the research standards are met during the execution of this study.

Sincerely,

Tafesse Matewos Karo(PhD)
Research and Technology
Transfer Vice President

ሀዋሳ ዩኒቨርሲቲ
የምርምርና ቴክኖሎጂ ሽግግር
ም/ፕሬዚዳንት ጽ/ቤት
Office of the V/President for Research and Technology Transfer
Hawassa University

P.O.Box: 5, Hawassa, Sidama, Ethiopia
Tel: +251-046-221-4210
+251-046-220-6107
Fax: +251-046-220-5041

Email:rttvp@hu.edu.et:

Websit: http://www.hu.edu.et.

ሁሌም ለልህቀት! *Ever to excel!*
